# A phenomenographic study exploring the conceptions of stakeholders on their teaching and learning roles in nursing education

**DOI:** 10.1186/s12909-022-03392-w

**Published:** 2022-05-26

**Authors:** Takaedza Munangatire, Patricia McInerney

**Affiliations:** 1 Department of Nursing, University of Namibia, P.O. Box 88, Rundu, Namibia; 2 Faculty of Health Sciences, Centre for Health Sciences Education, University of Witwatersrand, Johannesburg, South Africa

**Keywords:** Role, Phenomenography, Nurse educator, Nurses, Clinical instructor

## Abstract

**Background:**

Nursing education involves a number of stakeholders in the teaching and learning process, and these are student nurses, lecturers, clinical instructors and nurses. The role that each of these parties play in the teaching and learning process is dependent on each other and is key to the development of competence among student nurses. However, there is scanty literature on the discourse of how these stakeholders conceptualise their roles to maximise student learning. The objective of this study was to explore the conceptions of stakeholders on their teaching and learning roles in nursing education.

**Methods:**

Thirty-eight semi-structured interviews and three focus group discussions were conducted with nursing students, lecturers, clinical educators at a Namibian nursing college and nurses at teaching hospitals. Phenomenographic data analysis approach was applied.

**Results:**

Four conceptions were constructed that described the level of involvement of the different parties in teaching and learning. These were initiating, supporting, becoming part of and owning the teaching and learning role. Three dimensions of variation marked the differences among the conceptions: responsibility and accountability, priorities and level of collaboration.

**Conclusions:**

The parties involved in the teaching and learning in nursing education have qualitatively different understating of their roles and those of others. There is a pattern transcending from being at the periphery of teaching and learning to taking ownership of teaching and learning. And a movement from limited responsibility and collaboration to that of being responsible, accountable and high level of collaboration in the teaching and learning of nursing students. The conceptions in this study add more ways of enhancing collaboration between theoretical and clinical sites in nursing education, by ensuring that those involved are aware of their role and that of others and work collaboratively at the micro-level.

## Background

Nursing education involves a number of stakeholders in the teaching and learning process, and these are student nurses, lecturers, clinical instructors and nurses [1–3]. The role that each of these parties play in the teaching and learning process is dependent on each other and is key to the development of competence among student nurses [4]. The literature on how the different parties understand and perform their roles is lacking yet nursing education depends on the integration of theory and practice as facilitated by clinical and academic teachers [5, 6]. There are numerous calls for clarity on how the collaborative efforts among the stakeholders can be optimised [1, 7, 8]. The current study expands on the existing literature by exploring the conceptions of stakeholders on their teaching and learning roles in nursing education.

Globally there are variations and commonalties in how different stakeholders are involved in nursing education. Some European nursing literature showed that nurse educators assume at least three roles; an academic based at the university, a clinical educator in practice or teaching both in clinical practice and at university. In cases where the nurse educator works at the university only, they are complemented by clinical placement coordinators/clinical supervisors/clinical instructors [9]. Clinical instructors are employed by the academic institution and provide clinical teaching [10]. In addition to the nurse educators and clinical instructors, there are preceptors (nurses) who are employed by the hospitals for provision of nursing care but have a teaching role which they are either formally trained for or not [5]. At the center on these three educators, is the student who has a learning role that depends on the educators.

Teaching and learning process in nursing occurs in class, simulation and clinical settings [11] through an interactive process involving students, lecturers, clinical instructors and nurses [12]. However, each of the parties assume different roles in this process with competing and overlapping responsibilities. The literature showed that students, nurses, clinical instructors and lecturers have different experiences and understanding of their roles in teaching and learning process [13]. These experiences are shaped by the activities of the various parties as they engage in the teaching and learning process. Resultantly, the outcome of the nursing education process depends on how all the relevant stakeholders collaborate in their roles in relation to teaching and learning [14].

The need to deepen understanding on the roles of different stakeholders in teaching and learning in nursing is supported in literature. Specifically, examining conceptions through a phenomenographic approach will highlight differences in the stakeholders understanding and how the different ways of understanding are linked to each other. If these differences are known the stakeholders are able to relate better and help one another in the support of student learning [15]. According to Okoronkwo et al. [16] it is important to understand role expectations in the teaching and learning process. In nursing education academic staff, clinical nurses and students have responsibilities in the teaching and learning process hence all should be aware of the expectations of each other [17]. One suggested approach to improving role expectations in nursing education is collaborations such as academic clinical partnerships [18, 19]. Such collaborations create relationships and improves coordination among students, nurses and teachers which is necessary for better student learning outcomes [20, 21]. It is through teamwork between academic lecturers and clinical supervisors that theory can be integrated into practice [2, 7]. Phuma-Ngaiyaye et al. [5] argues that good communication among training institutions, preceptors and students is needed to ensure effective clinical teaching and learning. Despite this need for collaboration, clinical instructors feel they don’t get enough support from the lecturers and nurses [22]. Furthermore, little has been done to explore the nature of the collaboration that can maximise the effectiveness of each individual stakeholder’ s role in teaching and learning.

Existing literature has explored a number of issues around teaching and learning in nursing. Early studies have called for clarification of the roles of all stakeholders in nursing education [1, 9]. A study by Kristofferzon, Mårtensson, Mamhidir and Löfmark, [23] looked at nursing students' perceptions of clinical supervision with focus on various stakeholders and recommended that the roles of facilitators of teaching should be clarified. Some studies focused on clinical education or theoretical teaching in isolation yet nursing education is 50% theoretical and 50% practical [24]. One study looked at the role of preceptors, but neglected the role of the others [25]. Preceptors alone cannot lead to the success of the collaboration unless their efforts are complemented by other parties, hence the need to explore the conceptions of all the parties.

Lack of collaboration can contribute to poor outcome in team activities such as education of nursing students [26]. Bisholt et al., [27] argued that positive learning outcomes can be attained when nursing education staff, clinical staff and ward managers work together closely. However, there is evidence to suggest that the working relationships among preceptors, nurse teachers, ward managers, education providers and healthcare organisations are poor [27–29]. Subsequently, there are calls for a transformation of the way academic institutions and places of clinical practice work together [30]. Nevertheless, currently there is inadequate evidence to inform the transformation on how best collaboration can be done. There is even a bigger challenge where lack of collaboration is being associated with poor learning outcomes, without evidence to show that even if there is good collaboration learning outcomes would be different.

It can be argued that the focus on collaboration among stakeholders should not veil the important issue of teaching and learning. The overall goal of collaboration between lecturers and clinical teachers should be assimilating theory and practice to promote learning [2]. According to Pedregosa et al., [11] and Cant et al., [31] the roles of nurse educators, clinical teachers and the clinical managers should be complementary in a way that support clinical learning. Similar sentiments were expressed in studies by Panda et al., [32] and (Cervera-Gasch et al., [33] suggesting that the attitudes of nursing staff and clinical instructors towards their roles impact on student learning. These assertions from literature point to the need for research in the area of the roles of the different nursing education stakeholders and how these roles can ultimately lead to better learning outcomes. Researching on collaborations and roles outside the wider context of teaching and learning can result in a false believe of unproven potential benefits [34].

The emphasis on collaboration in nursing education is backed in literature. Several studies demonstrated that nursing students had positive learning outcomes when there was good relationship between ward managers and nurse educators [35–37]. Furthermore, Tuomikoski et al., [14] called on clinical teachers to engage academic institutions and other stakeholders if they desire to provide best clinical teaching and learning experiences. These arguments are indicative of the need for support among stakeholders involved in nursing education. A study by McLeod et al., [22] revealed that there was need to explore ways of supporting clinical teachers in their teaching role as some of their expectations are not met. In the same line, nursing students need the support and guidance from clinical teachers and nursing staff [38]. The role of clinical nurses in student learning is further supported in one study where nursing students’ satisfaction level was associated with involvement of clinical nurses in teaching. Regardless of this evidence, there remain a gap on how best all the different stakeholder in nursing education can collaborate creating a cohesive and supportive teaching and learning environment. McKenna et al., [39] added that there is an evidence gap in the organisation of clinical placements, one key area that can be addressed through collaborations among all nursing education stakeholders.

In an attempt to address the issue of collaboration, some clinical learning models have been proposed. Jayasekara, Smith, Hall et al., [12] evaluated the effectiveness of clinical education models for undergraduate nursing without delving into the roles of the various stakeholders in the implementation of the models. Although clinical education models have been found to improve clinical learning, they fall short of looking at nursing education holistically and clarifying the roles played by all parties in nursing education. Such models include Clinical supervision model, Preceptorship model and the Dedicated Education Model [3, 22]. In their evaluation of academic practice models, Pedregosa et al., [11] called for the analysis of how the different actors in nursing education are involved. Furthermore, Dube and Rakhudu, [25] study demonstrated that effective communication among preceptors, nurse educator and students was necessary for successful collaboration in preceptorship.

The evidence above on the clinical learning models suggest that more studies are needed. In particular, the success of collaborations in nursing education should be measured in terms of learning outcomes and not just creating a conducive environment. As researchers search more into this area, they should not neglect the most important aspects of teaching and learning. The teaching and learning of nursing students have been shown to be at different levels regardless of the level of collaboration. Early studies by Ironside et al., [40] and McNelis et al., [41] revealed that teachers and students put emphasis more on task completion as a measure of competence than the more complex components of learning the practice of nursing. These findings were confirmed in more recent studies which showed that nursing students had various conceptions of learning which varied from meeting curriculum demands to become a continuously developing professional [42]. Correspondingly, it has been reported that nursing students tend to focus on getting tasks done in less sophisticated conceptions of competence and shift their goal to attaining positive patient outcomes in more complex conceptions [43].

While this study is focused on the roles of stakeholders, these roles cannot be studied in isolation of the broad concept of teaching and learning. However, this study will not focus on conceptions of teaching and learning, but the conceptions of the roles will be discussed in the broad context of teaching and learning. In higher education it was reported that research on conceptions of teaching and learning may help in understand this subject better  [44]. In nursing education, conceptions were reported in different studies. Forbes, [13] studied clinical teachers conceptions of nursing and what the teachers focus on when teaching nursing students. Another study focused on the mentors’ conceptions of their role in facilitating clinical learning and found that collaboration among stakeholders was needed [1]. Furthermore, one study looked at the nurse educators’ conceptions of teaching showing three categories of transmitting knowledge, apprenticeship and facilitating ways of understanding [45]. The conceptions of teaching and learning in the above studies do not bring out how the roles of the different stakeholders’ interplay in the teaching and learning. Therefore, the purpose of this study was to understand the qualitative different ways in which stakeholders conceptualise their roles in nursing education. No studies have explored how the various parties in nursing education look at their roles and how these roles fit together despite the established need for effective collaboration between educational institutions and hospitals.

## Methods

### Design

This study applied phenomenography, a method aligned to qualitative research and explored the different ways in which people comprehend a phenomenon as well as how these various ways of understanding are interrelated [46, 47]. The method was developed in response to educational research needs at Göteborgs Universitet in Sweden [48]. Phenomenography seeks to understand people’s various conceptions of a particular phenomenon [49]. The study focused on how different stakeholders viewed their role and how their roles are related in the nursing education process which is in line with phenomenography which focus on variation and how those variations are connected [46]. In the description of their roles, the stakeholders will relate to their experiences of performing their role and this aligns to the assumption made by Marton [48], that people’s different ways of understanding a phenomenon are related to their experiences of the phenomena.

In this study, the roles of the stakeholders represent the phenomena under study and their conceptions of their roles will be explored. Conceptions represent experiences and perceptions of a collective and not individuals. Marton [49] described conceptions as a way of seeing or understanding something or what it means to a person. The conceptions are not associated with any particular group of participants but reflect broad range of perceptions and experiences participants have about a particular phenomenon. In nursing education, it is important to explore conceptions because they help reveal the different categories that shape a phenomenon. The sum of such categories makes up the results of the phonomyography study which is the “outcome space” [50]. These categories can form the basis of education, moving from the less inclusive conceptions to the more inclusive conceptions. The categories are hierarchical in nature and possess dimensions of variation which marks the difference between one category and the other [48, 51].

As a growing educational research tradition, phenomenography has been applied to study how teachers experience teaching [48, 52], their relationship with students [53] and diversity [54], how they perceive their work with parents and student teachers and how lecturers, preceptors and mentors conceptulised the relationships among them [40, 55].

### Setting

The researchers carried out this study at a nursing college and two teaching hospitals in Namibia. The college offered a diploma in nursing where theory and simulation classes were offered at the college and clinical education at the two teaching hospitals. A block release system (integration of theory and clinical practice) was used where students attend to theory and simulation for two weeks, then two weeks of clinical placement. The diploma was offered over three and half years culminating in the graduation of registered nurse midwives. Students learning in the class was mainly facilitated by the nurse educator, simulation facilitated by the clinical instructor and clinical learning mainly facilitated by the nurses with more support from clinical instructors and partially from the nurse educators. The nurse educators and clinical instructors were affiliated to the college as part the faculty while the nurses were employees of the hospital with a dual role of patient care and student teaching. The clinical instructors acted as the go between the college and the hospital communicating student placement allocations and objectives. Students had a responsibility of making sure their clinical registers are completed as evidence of learning. In the teaching hospital there were no dedicated teaching units neither was there any formalized model of clinical teaching and learning.

### Participants and sampling

Participants were purposively selected to maximise variation and ensure data saturation. While Stenfors-Hayes et al., [48] suggests that a sample size in a phenomegraphy study should be between 10 and 30 participants with a maximum variation, the nature of this study required a much larger sample size due to the different groups of participants. Fifty-nine participants were interviewed. These were twenty-one 2^nd^ year, students, ten 3^rd^ year students, ten 4^th^ year students, 4 lecturers, 4 clinical instructors, 10 nurses from two different hospitals departments (see summary in Table 1 below). The data were collected and analysed concurrently to ensure that conceptions of the participants were systematically discerned and data collection could stop when no significant conceptions were being generated [15, 56–58].

**Table 1:** Summary of participants

Participants	Data collection method
Second year students (21 with seven participants in each focus group)	Three focus group discussions
Third year students (10)	Semi structured interviews
Fourth year students (10)	Semi structured interviews
Lecturers and Clinical instructors (8)	Semi structured interviews
Nurses (10)	Semi structured interviews

### Data collection

Data were collected using in-depth semi-structured interviews because it is the recommended data collection method in phenomenography and suitable for generation of participant conceptions [48, 51]. TM conducted all the interviews. Interviews were conducted both telephonically and face to face. Face to face interviews took place in private rooms and all interviews were audio recorded lasting between 20 to 60 min. Data were collected from June 2018 to December 2018. An interview guide used was made up of the main questions asking participants their conceptions of their role in the teaching and learning and the role of the other players. Follow up questions were used to help participants clarify their points and add detail to their responses and provide concrete examples that supported their conceptions and to provide in depth response [59] The discussion was pursued until the participants indicated they had nothing more to share or when researcher felt enough data were collected to avoiding collecting too much data [60]. The questions used were;

- What do you do in your role as a lecturer/student/clinical instructor/nurse in the teaching and learning process?’
- What do you think should be your role in relation to others lecturer/student/clinical instructor/nurse?
- Can you give examples to demonstrated your role; Can you give examples to show how your role relates to the others in the teaching and learning process.

## Data analysis

Data were analysed using the seven steps of data analysis in phenomegraphic studies as outlined by Sjöström and Dahlgren, [61]. ATLAS.ti software was utilised to handle the data in the analysis process. TM transcribed the recorded interview audios verbatim. The detailed steps and process applied in the data analysis are outlined in the table below. The purpose of data analysis in phenomenography is not compare the conceptions of the different participants but to produce an outcome space detailing the qualitatively different ways in which participants experienced a phenomenon and explore how the experiences are related to each other [48]. Both researchers were involved in the data analysis and this is necessity in phenomegraphic data analysis so that preconceived ideas do not influence the process of analysis [62]. See Table 2 for the detailed step by step data analysis process.

**Table 2:** Steps in data analysis (Sjöström and Dahlgren, [61]

Transcription	TM transcribed all the interviews, getting an opportunity to start familiarisation with the data
Familiarisation consideration	Reading the interview transcripts to get initial impressions of the data. Both the researchers read the transcripts separately
Condensation	Identifying meaning units and generating codes. This was done separately and later codes and meaning units were compared. Consensus was agreed on the key codes
Comparison	The researchers scrutinised the meaning units to identify similarities and variations in the units
Grouping	The researchers allocated the meaning units into categories based on their similarities in relation to ways participants understood the phenomenon of role in teaching and learning
Articulating	Capturing the essential meaning of a certain category. The researchers read and discussed categories seeking to identify the essence of each category. It is at this stage where meaning units were further scrutinized to ensure that they exclusively fit into a category. In addition, the researchers sought for links among the categories, the hierarchy in the categories and the dimensions of variations were identified. This process was done iteratively until the researchers could not move any meaning units, consider different names or dimensions of variation
Labelling	Expressing the core meaning of the category Steps 3–6 are repeated in an iterative procedure to make sure that the similarities within and differences between categories are discerned and formulated in a distinct way
Contrasting	Comparing the categories through a contrastive procedure whereby the categories were described in terms of their individual meanings. The outcome space was viewed holistically verifying the order of the hierarchy of the categories. When consensus was reached on the key areas or hierarchy and dimensions of variation, the outcome space was produced

### Outcome space

**Fig. 1:**
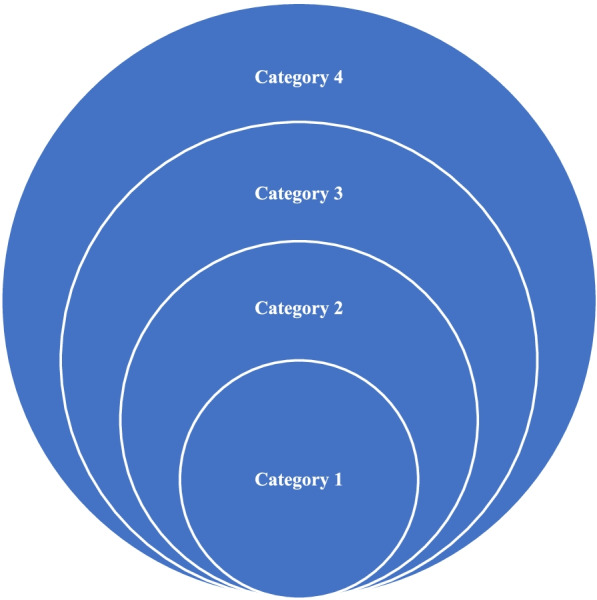
Categories of description

To understand the results of the data analysis in phenomenography studies, one should have a good picture of the outcome space in relation to the phenomena under study. The focus of phenomenography is exploring the qualitatively different ways in which people experience and understand the same phenomenon [63]. In this study the phenomena under study was the role of stakeholders and the question asked was “*What are the qualitatively different ways in which stakeholders in nursing education experience and understand their role in teaching and learning?*” According to Åkerlind [64] phenomenography looks at how different patterns of awareness and non-awareness of critical parts of a phenomenon result in variation in understanding. In the same way the data analysis focused on unveiling stakeholders’ patterns of understanding and non-understanding of their role in relation to teaching and learning. These conceptions generated a pattern that could be presented in categories which are hierarchically linked into an outcome space based on the variations in experiencing the critical aspects of the teaching and learning roles [53]. It has to be noted that what varies is not the expected roles of the stakeholders regarding teaching learning, but it is the way they experience these roles that vary [65]. The variations in the experiences of the stakeholders were reflected in what Marton and Booth [53] described as the internal and external horizons of awareness: “*to experience something in a particular way, not only do we have to discern it from its context [external horizon…but we also have to discern its parts, the way they relate to each other and the way that they relate to the whole [internal horizon]*. (p. 87). This means that the internal horizon refers to what is in focus, the internal relationship of the phenomenon’s parts to each other and its’ cohesive whole [53]. In this study the focus is on the role of each stakeholder, how the role of one stakeholder is related to that of others, how the role is related to teaching and learning, and how the role ultimately contributes to the outcome of teaching and learning. The external horizon refers to what is in the background of an experience [66]. The external horizon differs from person to person based on the critical aspects (dimensions of variation) of the phenomena they are focusing on. An individual with less sophisticated way of conception of a phenomenon has a narrow focus (internal horizon) and a wide background (external horizon) The diagram below shows this relationship. In category 1, the internal horizon is the first circle and the other three circles represents the external horizon. In category two, one has an expanded internal horizon where they are able to discern more aspects of the phenomena, but category three and four remains in their external horizon Fig. 1.

### Trustworthiness

The researchers took some measures to ensure trustworthiness. Firstly, to enhance generalisability, the researchers described in detail the context of the study, in particular how nursing education is delivered in Namibia and the parties involved including the different names that are used in different contexts [47]. Secondly reflexivity was applied throughout the research process as outlined by Sandbergh, [15] and its application was partly illustrated in the data analysis section above. The formulation of this research question was motivated by researchers interests in improving coordination among parties involved in nursing education. To support their motivation, researchers conducted intensive literature review on the teaching and learning to support the formulation of the research problem. In terms of selection of participants, a trained research assistant was used to recruit students and nurses. Lastly investigator triangulation was applied where the researchers engaged in continuous discussion and continuously revisited transcripts to ensure that the interpretations were supported with the data transcripts [67]. Credibility was ensured by outlining and showing the links between the conceptions and the data through the use quotations [59]. The outcome space of the study complied with criteria of phenomenographic outcome space which are each category demonstrating a peculiar way of experiencing or understanding a phenomenon. The categories were interlinked and the variation in the data was constituted by the only four categories (conceptions in this study) [68] .

## Findings

### Conception 1: Initiating learning

In this conception, the participants are in the periphery of the teaching and learning with their role almost non-existent. In their internal horizon they focus on their individual responsibility and no one takes the lead in collaborating learning especially in the clinical area. The idea of taking responsibility, becoming part of and owning the role of teaching and learning forms their external horizon. It is not clear who takes responsibility with all parties having different priorities. The student nurses felt the nurses, nurse educators and clinical instructors had a responsibility to initiate the process. On the other hand, the others expected the students to be self-directed and take responsibility of their in the clinical area.

*Sometimes you just don't know what to do so if you don't know what to do you are not learning anything because we need support. I think they must support us and tell us what to do and what to learn . They should show us and make us learn more things but some they let us just us… [Fourth year 9]*

*I think they do understand but I think on the other side they either take advantage of the clinical instructor because it seems like they tend to relax waiting for you to show them everything. When they go to the practical, they are not proactive, it’s like you owe them, to teach all the content. But it's not supposed to be like that, they should be kind of on the lookout to learn on their own rather than think like we have to teach everything to them ****(Clinical instructor 2).***

*Normally, I help the one who comes and asks, wanting to know. If you don’t come to me, I just leave you like that. Like others just come to show their faces around and do nothing ****(Nurse 5).***

Without anyone to take responsibility, a teaching and learning gap appeared where students did not act to learn waiting for the teachers and teachers waiting for the students. On the other hand, nurses were focused on delivering patient care and not prioritizing clinical teaching, clinical instructors and nurse educators focused on theoretical and simulation teaching respectively while students waited to be taught.

*So you see now for example now I'm assisting you as a students but then you don’t appreciate, students must understand that more than 50% of the learning in the clinical setup is for them they should know that facilitator are full of knowledge and of which we then they come with the right attitude and the right workmanship then also on the side of stuff we are able to help them (Nurse 1)*

*In addition, the nurses they expect you just to do things without necessarily showing you how they are done. So, I feel as much as they do (teach) us sometimes they can do more to help us, like more follow-ups and demonstrations on how to do the procedures ****(Third year 8).***

There was some kind of competition among the parties. For example, the lecturers and clinical instructors considered themselves as more knowledgeable and up to-date with nursing practice compared to nurses. The student nurses were sometimes caught in the midst of this competition and took sides on who is better among the teachers.

*Where is your clinical instructor, why didn’t your clinical instructor teach you? [Nurse 3]*

*Some of them (nurses) are not even happy they don't want to listen to students, I don’t know why. Maybe because they don't want to be seen doing wrong things; but our lecturers, there is no lecturer who is teaching you something which is wrong, that’s why sometimes you can go the extra mile to see what is being done in the current moment because they have to update us on what we should do ****(Second year 3).***

*Yes, just to be brought on to the same level with what we are doing because we learn every day, we do research, we Google, we learn every day about new things, but them they are more relying more on their experience ****(Lecturer 1).***

### Conception 2: Supporting teaching and learning

In this second conception the participants’ internal horizon expanded and focused not only on their responsibility in isolation of that of others, but opened up to initiating teaching and learning as well as supporting it. In the external horizon of the stakeholders lie the idea of becoming part of and owning the teaching and learning role. The different stakeholders’ support of learning focused on the parts rather than the whole process of student nurse teaching and learning. For example, the lecturer initiates the teaching and learning process by introducing students and then assign them tasks for further learning. However, the lecturer does provide students feedback on the given learning tasks.

*You have a lecturer in class she only gives a lecture on module one and then the rest of the module she will be giving you guys to present then after presentation she doesn't get back to you and to maybe just to summarise and tell you what is right and what is wrong just to go through the module with you guys [P7-FGD3]*

The nurses and the clinical instructors also took some responsibility for teaching but they felt there should be someone taking full responsibility or partial responsibility for the teaching role. Nurses suggested that they were only getting involved in the teaching and learning when left with no choice but to teach the students.

*Like I said earlier, we just need to have someone with the responsibility of teaching the students only because you find yourself in a situation when there is the clinical instructor should come always to teach the students with the procedures but if the students keep coming to us you are forced to create time for them [Nurse 3]*

*As a clinical instructor I don’t teach much, I am more into simulating the procedure, showing students how it’s done. I provide students with tools of doing the procedure hence they follow as I demonstrated. I also give them time to simulate to each other and when they go to the clinical area, they have to put it into practice. [Clinical instructor 3]*

In general, the role of teaching and learning beyond an individual stakeholder’s responsibility is considered a secondary priority. The collaboration of teaching and learning appear to be the one where one has to cover up gaps left by the other part if they have the opportunity to do so.

### Conception 3: Moving towards a holistic role in the teaching and learning

Under this conception, the internal horizon is further expanded and the stakeholders’ focus was on their role as part of the whole teaching and learning process rather than individual isolated role. The external horizon was made up of one taking ownership of the teaching and learning role. The parties began to recognise the need for themselves and the need for others to do more to facilitate teaching and learning. The stakeholders started to see or have a desire to know the roles from the other sides’ perspective. For example, nurses wanted to understand the role of the lecturers and clinical instructors. There was a shift towards taking responsibility of teaching.

*It is not adequate at all for the lecturer or clinical instructor to come in, do the procedures, and show students how things are supposed to be done. That is making the job difficult for us because we want to be part of the teaching and know the side of the lectures and instructors because helping students in the clinical area is our responsibility (Nurse 7).*

I *can say being a lecturer like in our institution a lecturer is having like 65 percentage theory in the class not a hundred percent let me say 85% because there are also procedures being taught by the clinical instructor meaning the lecturer is responsible for the main content to be taught to the students but also should then provides like that 35% in the clinical area just to make sure the theory and practical are linked [Lecturer 1]*

Teaching and learning became an equally important priority rather than an extra priority. This is more pronounced among the nurses as they looked at teaching and patient care as their priorities.

*Now you have to provide nursing care and you also to teach the students, how can you do the other without neglecting the other. So, I have to like call them come and see how we catheterize, come and see how we put a nasogastric tube. Come and try this while I watch you [Nurse 2]*

The conceptions showed that there were moves towards consolidating the roles of all parties discussing how their roles can be integrated in the teaching and learning of student nurses.

*On some occasions, we call for clinical meetings to try to deliberate on issues affecting the learners. Students are given an opportunity to share their views on how they see the learning process. The nurse managers and lecturers also attend. I think we are getting there (Nurse 4).*

*The clinical instructors can work more together with the nurses in the wards so that they can see where the students are lacking because the nurses understand more where the students are lacking. So, the clinical instructors make the effort to find where they can improve. So, by going to the hospital that can make very good improvement and as students we can do our part without having to try and please the lectures or the nurses (Fourth year 5).*

*I am thinking, I teach the students when the clinical instructor is not there, so when the clinical instructor is there, they have to come to me and we see how we can amalgamate the theory and practice as well as our efforts in helping the students… [Nurse 1]*

### Conception 4: Owning the role of teaching and learning

In this conception, the internal horizon of the stakeholders expanded to be aware of their role as that of not only initiating, supporting and becoming part of teaching and learning to that of completely owning the role. At this stage all the stakeholders were aware of all the critical aspects in the collaboration of teaching and learning in nursing education. In their external horizon could be other aspects that can improve the collaboration. The parties began to recognize the need to do more than their primary part in facilitating learning. There is a resemblance of team work and acceptance of each other as equal partners in the teaching and learning process. Each part acknowledges their role and that of others in the teaching and learning process of student nurses. Nurses consider themselves as key in clinical teaching and creating learning opportunities for students and linking up with lectures and clinical instructors.

*Therefore, I have to be a role model and play a leading role and create opportunity for the students to exercise the skills that they are due to learn in the clinical area. At the same time, we also assess in the end of it all, after assessing we also award marks give the marks to the students [Nurse 5]*

*We communicate back to the educators to say yes, we have student XYZ and this is how they've performed and this is how they perform in particular areas so they are devoid of information in this area. Therefore, we are facilitators of the learning exercise in the clinical area [Nurse 7]*

*As students we make sure that we do more, not just wait to be spoon-fed should also do more on our subjects that would help us [P1-FGD3]*

The lecturers equally accept that what they teach is equally important as to what is taught in the clinical area hence don’t see their role as different from the clinical instructors and nurses.

*I don't see my role different from the clinical instructors or the nurses in practice because that is what I always tell my students as well that the theory and the practice is to be linked together what I'm teaching them in theory that is what they are supposed to go and carry out in their clinical [Lecturer 4]*

In the same line students recognise their role and are willing to take charge of their learning. The stakeholders begin to see their roles as complementary in the teaching and learning of students.

*The students make sure that we do more, not just wait to be spoon-fed should also do more on our subjects that would help us. In the end we are accountable for our learning as much as the teachers are accountable for their role [P1-FGD3*

*While delivering nursing care of the patient comes first, I cannot say teaching students is secondary because when you are teaching in the clinical area you are teaching on patients so what I will be doing is teaching while I am actually rendering care to the patient. I feel I have to make sure that students learn , I am answerable to my profession to mentor these students [Nurse 3]*

*I think so we do understand each otherwhatever we are doing here actually starts in the class and when the students come here, we are not starting to teach them, we are continuing what they have started with the lecturers. So, I think we have the same understanding and can say we are complementing each other [Nurse 6]*

## Discussion

### Summary of findings

This study revealed that stakeholders involved in teaching and learning of nursing students understand their roles in a qualitatively different way. At the basic level of understanding, the different stakeholders considered teaching and learning as a task to be initiated by the other part and each do their own part in isolation. At a more sophisticated category conceptions teaching and learning of nursing students was understood as a shared responsibility to be prioritised and carried out in an integrated and complementary manner. These conceptions mean that in the teaching and learning of nursing students, some may understand their role in a less sophisticated way, while others in a more sophisticated way. The conceptions are not fixed as people can develop from less complex to more complex conception and depending on circumstances some may have actions consistent with less sophisticated conceptions even though they reached the most sophisticated conceptions. Challenges also arise when stakeholders with different conceptions of their roles have to work together. Table 3 above is the summary of the findings showing the conceptions and the dimensions of variation which are discussed in detail below.

**Table 3:** Outcome space

		**Dimensions of variation**		
		**Responsibility and accountability**	**Priorities**	**Level of collaboration**
**Conceptions**	1.**Initiating**	No one takes responsibility for initiating teaching and learning	Parties have different priorities regarding teaching and learning	Competing
	**2. Supporting**	Takes some responsibility for teaching and learning	Teaching and learning is considered a secondary priority	Covering gaps left by others when there is an opportunity
	**3.** **Becoming part of**	Takes full responsibility for teaching and learning without being accountable	Teaching and learning becomes an equal priority with other responsibilities	Consolidating efforts with parties reaching out to enhance the teaching and learning
	**4.** **Owning the role**	Becomes accountable	Teaching and learning becomes of core priority	Complementary efforts with the different parties working together effectively

### What the study adds to literature

The role of lecturers, clinical instructors, nurses and nursing students have been reported in the literature. However, this has been done in parts and not from a holistic perspective focusing on all parties and their roles. Also, no study has profiled the conceptions of the roles of these stakeholders as done in this phenomenography study. While this is not the first study to examine the roles of the different players involved in the process of nursing education. The study brought all the four key players in one study and triangulated their conceptions regarding their role in the education process. By using a phenomegraphic approach, the study exposed different levels of understanding or the different stakeholders in the education of nurses and how these result in either contradictions or collaboration. The conceptions in this study could provide an explanation on why the collaboration among health institutions, and academic institutions have not always been successful. Those directly involved in the teaching and learning of nursing students do not always view and comprehend their roles in a complementary and collaborative way [10, 20, 69].

### Least sophisticated conception of teaching and learning role

Collaborations between faculty and clinical area for teaching and learning have been proposed as the key to effective nursing education [1, 9, 23]. This study recognise the need for such collaborations but argue that the collaboration should occur both at macro and micro level where the individual parties fully understand their role and that of other parties. Low level conceptions in this study suggested that the parties involved in teaching and learning do not conceptualise their roles and that of others in the expected manner. Subsequently the parties have competing priorities, are in competition and not willing to fully shoulder the teaching and learning responsibility. While nurse educators and clinical instructors would teach by default theoretically and in simulation, clinical teaching did not come naturally. Subsequently when it comes to clinical teaching, its left mainly to the nurses who unfortunately prioritised patient care over teaching students [20, 70]. The hesitancy to provide clinical learning confirms the faculty expectation that students should be self-directed and accountable for attaining their learning outcomes [71]. Although the concessions by Bvumbe [17] that, clinical instructors and lectures can’t be in clinical sites all the time when needed for clinical teaching is valid, it may not be used as a weakness but an opportunity for greater collaboration with the clinical staff. Nurse educators generally have a dual role, which is theoretical and clinical teaching although they do not always apply this [72].

The competing actions revealed in the conceptions are reflected in several literature. There is a theory -practice gap, with nurses being clinically competent but lacking teaching skills and nurse educators being competent as teachers but lacking clinical skills [73, 74]. There are call for both sides to support each other, for example AlMekkawi et al., [75] indicated that faculty needed clinical skills and the nurses needed teaching support [76]. However, this is not possible if their conceptions are oriented towards competition rather than collaboration as revealed in the study. The reported poor collaboration in literature cannot be improved if the conceptions of the different parties are at a low level [17].

At a relatively advanced but still low conception, the different parties see their roles as supporting the other parties in teaching with none take full ownership. In some studies, it was reported that nurses accept their role of teaching students but they do not always consider it as a priority or fail to fully execute this role [25, 73, 77]. The failure to priorities or takes full responsibility of teaching by nurses has been attributed to lack of required teaching skills [78]. On the other hand, nurse educators leave clinical teaching for the clinical nurses and act as a link between clinical and theoretical teaching only [5].

### Most sophisticated conception of teaching and learning

While ccollaborations between academic institutions and clinical settings have been reported to improve student learning, the collaboration has been reported at a macro level [79]. This study explored the collaboration at a micro level revealing the different levels at which the collaborations work well and where they fail. While it is expected that all parties involved in teaching and learning accept and assume their role, the conceptions in this study showed that the parties are at different levels of understanding their roles. Clinton et al., [80] supports this finding who reported that students gradually assume responsibility of their learning as their understanding develops. Similar scenarios are displayed in nurses who struggle to balance their patient care responsibility and students teaching [81]. This study showed that once the parties reach a certain level of understanding, teaching and practice are considered equal priorities facilitating the nurses to take full responsibility of teaching. At this level all parties start to form meaningful collaborations that support student learning holistically.

Key stakeholders involved in the education of nurses should thrive to engage at the most sophisticated conception that allows students to be self-directed, roles of teachers to be complementary within a framework of mutual respect. These conceptions support the assertion by Tuppal et al., [82] that the success of education depends on nursing faculty members, nursing managers, nurses, and nursing students’ contribution towards working together. There should be a move away from nurse educators only recognising nurses as key part of the teaching process because they are overworked and cannot cover clinical teaching [83]. The expectation for students to be self-directed is legitimate however students need to be supported in the development of taking responsibility of their learning [74]. It has been reported that students need constant support and reminders of their task as well as directing them on how to learn since most only come to understand their full responsibility late in their studies [84, 84]. The responsibility of teaching and learning should be shouldered equally by the teachers and learners in a complementary partnership with both parties being held accountable for the outcome of the teaching and learning process [81]. Based on the findings of this study, the argument is that only when the parties involved in teaching and learning have reached the most sophisticated conception of their role will there be clear roles, collaboration and desire to be responsible and accountable for learning [85].

### Limitations

This study was conducted at one nursing college making the findings contextual, however the findings linked with literature from other settings. While it has been argued that researchers should step aside and apply reflexivity in the research process, researchers in this study confirm that while the conceptions were based on the words of the participants, these words were influenced by researchers during data collection and the interpretation were driven by the philosophies and interest of researcher. With researchers getting involved data cannot interpret itself and interpretations cannot be completely without bias of researchers as they are the once who construct the conceptions. Therefore, readers and other researchers are open to scructinise the conceptions based on the data given and arrive at a different interpretation. Further researcher is recommended to further extend our understanding of the complex nursing education process in the eyes of the concerned stakeholders.

However, in reality, this is not practical, the researchers undertook this study because of their interest in the topic and reflexivity does not take that away. When analysing the data, researchers use their own interpretations of the data, but only retain those interpretations that can be supported by the data. Assuming that reflexivity distances researchers from any biases, is denying that, the point of reference for data analysis are the researchers’ knowledge, philosophies, their thoughts, experiences and feelings in relation to the data. These form basis of the data interpretation and analysis (without which no analysis can take place), which through due diligence and reflexivity, researchers then shape to reflect on the data and be presented as the findings. Marton and Booth [51], and Marton [48] suggested that the categories at the end of the data analysis relate the participants as a group rather than individuals. It can be argued that this group includes the researchers as they conceptulised the research, participated and shaped the interview as well as the data. While their voice may not be explicit in the participants transcripts, the researchers do influence the participants' voice.

## Conclusion

The parties involved in the teaching and learning in nursing education have qualitatively different understating of their roles and those of others. There is a pattern transcending from being at the periphery of teaching and learning to taking ownership of teaching and learning. And a movement from limited responsibility and collaboration to that of being responsible, accountable and high level of collaboration in the teaching and learning of nursing students. The conceptions in this study adds more ways of enhancing collaboration between theoretical and clinical sites in nursing education, by ensuring that those involved are aware of their role and that of others and work collaboratively at the micro level.

## Data Availability

The datasets generated and/or analysed during the current study are not publicly available due to participant privacy, but are available from the corresponding author upon reasonable request and approval by the ethical bodies.
